# Study on the multi-lane lattice model for highways based on a novel lane-changing mechanism

**DOI:** 10.1016/j.heliyon.2024.e33262

**Published:** 2024-06-18

**Authors:** Yi-rong Kang, Chuan Tian

**Affiliations:** a School of Transportation Engineering, Guizhou Institute of Technology, Guiyang, 550003, China; b School of Economics and Finance, Guizhou University of Commerce, Guiyang, 550014, China

**Keywords:** Multi-lane lattice model, Lane-changing rules, Aggressive lane-changing behavior, Stability conditions, Highways

## Abstract

This paper reconstructs the lane-changing rules of a multi-lane highway system based on the aggressive lane-changing characteristics of actual drivers. Based on these observations, a new multi-lane lattice model is proposed. The linear stability conditions for the new model are derived. The density wave equation of the novel model is generated by exploring the evolutionary law of traffic congestion in the multi-lane highway system near the critical stability point. The correctness of the theoretical analysis results is verified using numerical simulations. The results of the study show that for a given number of multi-lane systems, the stability of the traffic flow is positively correlated with the driver's aggressiveness in changing lanes. In particular, when the adjustment intensity coefficient reaches 1, the stability of the traffic flow is optimal. On the other hand, when the lane-changing aggressiveness coefficient is kept constant, the stability of the multi-lane system increases as the number of lanes gradually increases from 1 to 4. Furthermore, the findings demonstrate that the propagation range and the size of flow fluctuations can be substantially reduced by increasing the aggressiveness of lane changes as well as by increasing the number of lanes.

## Highlights

• Based on aggressive lane-changing characteristics, a novel multi-lane lattice traffic flow model for highway is proposed.
• Linear and nonlinear stability analysis have been conducted.
• Theoretical results are validated through simulation.
• The findings indicate that multi-lane flow can be sustained stable by increasing lanes and the aggressiveness of lane changes.

## 1 Introduction

Traffic congestion has become a serious social problem in terms of transport efficiency, and it has attracted much attention in the past decades. Many different traffic flow models have been constructed to reveal the mechanism of traffic congestion. In terms of modeling scale, traffic flow models can be divided into two categories: microscopic traffic flow models and macroscopic traffic flow models. Microscopic traffic flow models focus on describing the movement of individual vehicles in the road system, and are represented by the car-following model [[1], [2], [3], [4], [5], [6], [7]] and the cellular automata model [8,9]. Macroscopic traffic flow models are concerned with the dynamic evolution of traffic flow as a whole. Its representative models are mainly the continuum model [[10], [11], [12]] and the lattice model [13,14]. Among these models, the single-lane lattice model proposed by Nagatani [13] in 1998 has received extensive attention from scholars due to its effective combination of the advantages of both macro- and microscopic traffic flow models. Further, Nagatani [14] extended his single-lane lattice model to a two-lane framework by introducing the factor of vehicle lane-changing behavior. Subsequently, a series of expanded traffic flow lattice models were proposed in succession through the introduction of various factors in the transportation system, the details of which are shown in Table 1.

**Table 1:** Extensions and derivations of the lattice traffic flow model.

Authors	Characteristics	References
Wang, Kaur, Peng	Driver's predictive effect	[[15], [16], [17], [18], [19]]
Redhu and Siwach	Traffic jerk	[20]
Jin, Wang	Curved road with passing	[21,22]
Peng and Kuang	Honk effect	[23,24]
Wang, Ge, Qi	“Backward looking” effect	[[25], [26], [27]]
Mei, Zhang, Jiang	On-ramp and off-ramp	[[28], [29], [30]]
Long, Li	Flux limit effect	[31,32]
Mei, Peng, Zhang	Self-stabilization effect	[28,33,34]
Wang, Li,Zhai	Driver's memory effect	[[35], [36], [37], [38]]
Zhang, Peng, Pan,Zhao	Delayed-time effect	[[39], [40], [41], [42], [43], [44]]
Zhai and Peng	Cyber-attacks effect	[[45], [46], [47]]
Sharma, Peng, Zhang	Driver's characteristics	[[48], [49], [50]]
Peng, Redhu, Wang	Traffic interruption probability	[[51], [52], [53]]
Zhu, Sun	Lane-changing rate	[[54], [55], [56]]
Yang, Peng	Optimal flux difference estimation	[57,58]
Tian, Peng	Optimal current difference	[59,60]
Kang, Ge	Driver's physical delay	[61,62]
Zhao	historical current integration effect	[63]

Despite the extensive and in-depth study of traffic lattice models, the models that have been developed are mainly focused on single-lane or two-lane system scenarios. In fact, it is well known that multi-lane systems, where highways consist of more than two lanes, are widespread on the majority of highways. However, there are very few traffic flow lattice models that have been systematically studied for multi-lane systems. Very recently, Madaan and Sharma [64] expanded the traffic flow lattice model for multi-lane systems with optimal current difference by utilizing the concept of two-lane systems in order to increase the applicability of the lattice model for real-world scenarios. Later, in 2022, Madaan and Sharma [65] further investigated the impact of delayed feedback control on multi-lane systems. In 2023, Zhai et al. [66] proposed a new multi-lane lattice hydrodynamic model considering the passing effect. The results of the aforementioned multi-lane lattice models show that multi-lane systems are very effective in suppressing traffic congestion and optimizing traffic flow by properly incorporating the effect of lane-changing behavior.

**Fig. 1:**
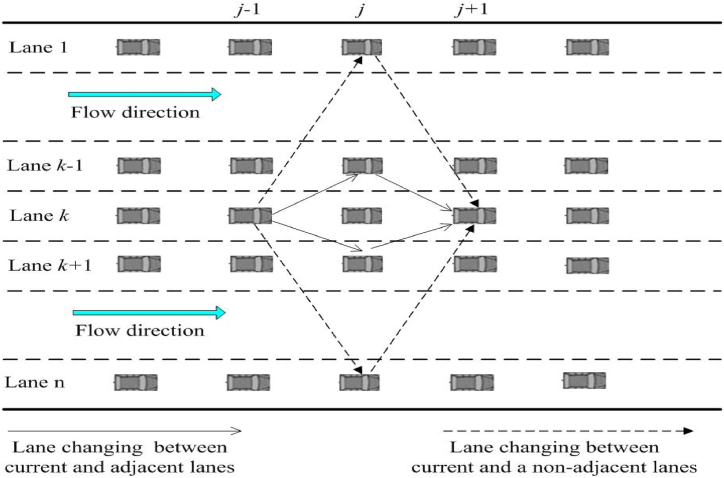
Schematic diagram of a multi-lane highway with lane-changing behavior.

In recent years, the augmentation of vehicular motion processes by traffic cyber-physical systems (T-CPS) has garnered significant attention from researchers [[67], [68], [69]]. Enabled by T-CPS technology, drivers' perceptual boundaries have been vastly broadened, and vehicle-to-vehicle information exchange has reached unprecedented levels of smoothness. This advancement enables road system vehicles to perform intricate collaborative tasks. As depicted in Fig. 1, in a multi-lane system equipped with sophisticated T-CPS systems, drivers can access real-time environmental data from multiple surrounding lanes. Consequently, lane-changing behavior is no longer confined to traditional adjacent lane switching, allowing for more flexible multi-lane maneuvers. This shift complicates and diversifies the lane-changing mechanism in multi-lane systems compared to two-lane systems, having a more profound impact on overall traffic flow. Nevertheless, existing multi-lane lattice models still fall short of adequately describing and modeling such intricate lane-changing patterns. In the current multi-lane traffic flow lattice models [19,[64], [65], [66]], the basic assumption of the lane-changing rule is that the density of the traffic flux in the neighboring front lattice site on the target lane only needs to be smaller than the density of its neighboring front lattice site on the current lane (i.e., the lateral distance between the current vehicle and its nearest vehicle in the target lane only needs to be larger than the headway between the current vehicle and its nearest vehicle on the current lane), and then lane-changing behavior is likely to occur. Obviously, this lane-changing assumption is overly simplistic and does not align with actual traffic conditions. This condition suggests that a lane-changing maneuver could occur even when the lateral distance to a vehicle in the target lane is marginally greater than the headway, implying frequent lane changes. However, this approach fails to consider the safety spacing constraint that is crucial for safe lane-changing. In reality, a situation where the lateral distance in the target lane is slightly larger than the headway of the vehicle in question is insufficient to motivate a driver to make a lane-changing decision for safety reasons. Therefore, it is evident that a systematic refinement of the lane-changing behavior in existing multi-lane lattice models is necessary to accurately reflect actual traffic scenarios. In view of this, in order to compensate for the shortcomings of the current multi-lane system in the study of lane-changing rules, this paper comprehensively reconstructs the lane-changing rules of the multi-lane highway system, based on the aggressive lane-changing characteristics of actual drivers. On this basis, we derived a new vehicle conservation equation using the basic principle of lattice models, and established a new multi-lane lattice model based on it. Subsequently, we carried out theoretical analyses and numerical simulation studies on the new model. This innovative work aims to more accurately simulate and analyze the vehicle behavior in multi-lane systems, in the hope that it will provide an effective analytical model for us to accurately understand and grasp the evolution law of macroscopic traffic flow in multi-lane systems.

In Section 2, for a highway scenario containing *n* lanes, based on the new lane-changing assumption, we reconstruct the continuity equations of the lattice model and thus propose a new multi-lane lattice model. In Sections 3, 4, we theoretically analyze the new model, explore the linear stability and nonlinear properties, respectively, and obtain the linear stability condition and the density wave equation describing the congestion propagation law in the multi-vehicle highway system. In Section 5, we carry out numerical simulation studies of the new model under periodic boundary conditions and check the correctness of the theoretical analysis. In Section 6, the conclusions of the study in this paper are given.

## 2 Models

In 2021, Madaan and Sharma [64] extended the two-lane lattice model to a multi-lane highway system with *n* lanes. In this multi-lane system, vehicles can freely change lanes between these *n* lanes. Since all lanes are homogeneous, the probability of a vehicle choosing a lane is equal. By drawing on the modeling mechanism of the two-lane lattice model, Madaan and Sharma [64] proposed the following assumptions to construct a lattice model suitable for multi-lane highways:

1) The vehicles in $k^{th}$ lane will be conserved except the lane changing, where 1≤k≤n.
2) If the vehicles density at site j−1 of $k^{th}$ lane is greater than site j on $m^{th}$ lane, where m∈[1,n] and m≠k, then lane-changing behavior may occurs from lane k to lane m, with the lane-changing rate $\gamma \left|\rho_{0}^{2}V^{\prime }\left(\rho_{0}\right)\right|\left(\rho_{k,j-1}-\rho_{m,j}\right)$.
3) If the vehicles density at site j of $m^{th}$ lane is greater than site j+1 on $k^{th}$ lane, where m≠k, then lane-changing behavior may occurs from lane m to lane k, with the lane-changing rate $\gamma \left|\rho_{0}^{2}V^{\prime }\left(\rho_{0}\right)\right|\left(\rho_{m,j}-\rho_{k,j+1}\right)$.

Based on the above assumptions, the general continuity equation for $k^{th}$ lane on highway is given as

(1) $$\partial_{t}\rho_{k,j}+\rho_{0}\left(\rho_{k,j}v_{k,j}-\rho_{k,j-1}v_{k,j-1}\right)=\gamma \left|\rho_{0}^{2}V^{\prime }\left(\rho_{0}\right)\right|\left(\sum_{\begin{array}{c} m=1\\ m\neq k \end{array}}^{n}\rho_{m,j+1}+\sum_{\begin{array}{c} m=1\\ m\neq k \end{array}}^{n}\rho_{m,j-1}-2\left(n-1\right)\rho_{k,j}\right)$$

where $\rho_{k,j}$ and $v_{k,j}$ represents the density and velocity of $k^{th}$ lane at site j, respectively. And n is the total number of lanes in a highway system. Here, γ is dimensionless lane-changing rate constant coefficient. $\left|\rho_{0}^{2}V^{\prime }\left(\rho_{0}\right)\right|$ is introduced to ensure that γ is a dimensionless parameter.

By mapping above Eq. (1)∀k into single lane system, one can obtain the following continuity equation [64]:

(2) $$\partial_{t}\rho_{j}+\rho_{0}\left(\rho_{j}v_{j}-\rho_{j-1}v_{j-1}\right)=\gamma \left(n-1\right)\left|\rho_{0}^{2}V^{\prime }\left(\rho_{0}\right)\right|\left[\rho_{j-1}+\rho_{j+1}-2\rho_{j}\right]$$

Where $\rho_{j}=\frac{1}{n}\sum_{k=1}^{n}\rho_{k,j}$, $\rho_{j}v_{j}=\frac{1}{n}\sum_{k=1}^{n}\rho_{k,j}v_{k,j}$.The evolution equation in References [64] used for the multi-lane system is as follows:

(3) $$\rho_{j}\left(t+\tau \right)v_{j}\left(t+\tau \right)=\rho_{0}V\left(\rho_{j+1}\left(t\right)\right)+\lambda \left[\rho_{0}V\left(\rho_{j+2}\left(t\right)\right)-\rho_{0}V\left(\rho_{j+1}\left(t\right)\right)\right]$$

where the parameter τ represents the delay time, which is inversely related to the driver sensitivity a, i.e. τ=1/a.Term $\rho_{0}V\left(\rho_{j+2}\left(t\right)\right)-\rho_{0}V\left(\rho_{j+1}\left(t\right)\right)$ denotes the optimal current difference (OCD) information on site *j* + 1 at time *t*. λ is the reaction coefficient of OCD. Parameter $\rho_{0}$ is the average density of the multi-lane highway system. V is the optimized velocity function. In the classical lattice model, the function equation for optimized velocity is as follows [14]:

(4) $$V\left(\rho \right)=\tanh\left(\frac{2}{\rho_{0}}-\frac{\rho }{\rho_{0}^{2}}-\frac{1}{\rho_{c}}\right)+\tanh\left(\frac{1}{\rho_{c}}\right)$$

where $\rho_{c}=0.25$ represents the critical density value. When $\rho_{0}=\rho_{c}$, Eq. (4) has a inflection point at $\rho =\rho_{c}$.

In real multi-lane highway system scenarios, when different vehicle densities are present in two adjacent or non-adjacent lanes, the driver may have a motivation to change lanes, but this does not mean that he/she can actually carry out a lane change. This is because the constraints of safe driving conditions also have to be considered. However, in the multi-lane lattice model described in References [64] and its subsequent improvements [19,65,66], the assumed lane-changing condition is so extreme that lane-changing behavior occurs only if the vehicle density at site j−1 on lane k is just higher than that at site j on the target lane m, where m≠k. In fact, this is a very aggressive assumption for lane-changing, especially when the density of vehicles on all lanes is high, and such lane-changing behavior would likely lead to potential collisions between vehicles. Therefore, in real driving situations, such lane-changing behavior does not occur universally. Improvements to the lane-changing mechanism and the resulting continuity equations for multi-lane systems are thus required. Recently, Li et al. [70] devised a more rational lane-changing rule for a two-lane system, assuming that lane-changing behavior occurs just only when the lateral distance of the current vehicle in the target lane is much larger than its preceding headway, in many cases, a multiple of the preceding headway. Meanwhile, based on the multiplicative relationship between the headway and the lateral distance, a parameter characterizing the aggressiveness of the driver's lane changing was constructed. The results show that, based on this lane-changing assumption, the degree of driver lane-changing aggressiveness has an important influence on the stability of the traffic flow in the two-lane system. However, unfortunately, the lane-changing mechanism suggested by Li et al. [70] was not addressed in the framework of multi-lane systems.

In order to make the multi-lane lattice model closer to traffic flow reality, we improve the multi-lane system lattice model by borrowing the design viewpoints of vehicle lane changing mechanisms from Li et al. [70] Therefore, we assume that the lane-changing behaviors will occur with the rate $\gamma \left|\rho_{0}^{2}V^{\prime }\left(\rho_{0}\right)\right|\left(\rho_{k,j-1}-f\rho_{m,j}\right)$ from lane k to lane m if the density at site j−1 on lane k is higher than f times density at site j on lane m, where f represents the adjustment intensity coefficient for driver lane-change aggressiveness. It should be noted that the larger the value of f, the lower the density of vehicles at site j in the target lane compared to the density at site j−1 of the current lane, which indicates that the lateral distance for the considered vehicle in the target lane is much larger than its headway in the current lane, and the easier it is for the lane-changing process to be realized safely. In other words, the value of the adjustment intensity coefficient f characterizes the aggressiveness of the driver's lane-changing process, the larger the value, the less aggressive (i.e., the more conservative) the driver's lane-changing process is, and the smaller the value, the more aggressive the driver's lane-changing is.

Similarly, when the density of site j on lane m is f times higher than the density of site j+1 on lane k, lane-changing behavior will occur, and the resulting lane-changing flow rate is $\gamma \left|\rho_{0}^{2}V^{\prime }\left(\rho_{0}\right)\right|\left(\rho_{m,j}-f\rho_{k,j+1}\right)$. Based on the above assumptions of the new lane-changing rule, the total inflow from the neighboring lattice site j−1 on other lanes m (m≠k) to the lattice site j on the lane k is $\sum_{\begin{array}{l} m=1\\ m\neq r \end{array}}^{n}\gamma \left|\rho_{0}^{2}V^{\prime }\left(\rho_{0}\right)\right|\left(\rho_{m,j-1}-f\rho_{r,j}\right)$, and the total outflow from the lattice site j on lane k to the lattice point j+1 on each of the other lanes m (m≠k) is $\sum_{\begin{array}{l} m=1\\ m\neq r \end{array}}^{n}\gamma \left|\rho_{0}^{2}V^{\prime }\left(\rho_{0}\right)\right|\left(\rho_{r,j}-f\rho_{m,j+1}\right)$. Thus for site j on lane k, the total change in vehicle density due to the lane-changing is $\gamma \left|\rho_{0}^{2}V^{\prime }\left(\rho_{0}\right)\right|\sum_{\begin{array}{l} m=1\\ m\neq r \end{array}}^{n}\left(\rho_{m,j-1}\right)-\left(n-1\right)\left(f+1\right)\rho_{r,j}+f\sum_{\begin{array}{l} m=1\\ m\neq r \end{array}}^{n}\left(\rho_{m,j+1}\right)$. Hence, the general continuity equation for $k^{th}$ lane in n-lane highway system can be formulated as following:

(5) $$\partial_{t}\rho_{k,j}+\rho_{0}\left(\rho_{k,j}v_{r,j}-\rho_{k,j-1}v_{k,j-1}\right)=\gamma \left|\rho_{0}^{2}V^{\prime }\left(\rho_{0}\right)\right|\left(\sum_{\begin{array}{c} m=1\\ m\neq k \end{array}}^{n}\rho_{m,j-1}+f\sum_{\begin{array}{c} m=1\\ m\neq k \end{array}}^{n}\rho_{m,j+1}-\left(f+1\right)\left(n-1\right)\rho_{k,j}\right)$$

By iterating k from the natural number 1 to n, then adding the left and right sides of Eq. (5), a new continuity equation for the n-lane highway traffic system can be obtained as follows:

(6) $$\partial_{t}\rho_{j}+\rho_{0}\left(\rho_{j}v_{j}-\rho_{j-1}v_{j-1}\right)=\gamma \left(n-1\right)\left|\rho_{0}^{2}V^{\prime }\left(\rho_{0}\right)\right|\left[\rho_{j-1}+f\rho_{j+1}-\left(f+1\right)\rho_{j}\right]$$

It is assumed, in accordance with Nagatani's concept in References [14], that the lane-changing behavior has no bearing on the evolution equations for the traffic flow on each lane. Therefore, in order to concentrate on investigating the effects of the number of lanes and driver lane-changing aggressiveness characteristics on the dynamical properties of the traffic flow, we employ the following evolutionary equations that Nagataniti proposed in a two-lane system.

(7) $$\rho_{j}\left(t+\tau \right)v_{j}\left(t+\tau \right)=\rho_{0}V\left(\rho_{j+1}\left(t\right)\right)$$

Consequently, we derive a novel multi-lane traffic flow lattice model comprising equations (6), (7) that consider drivers' aggressive lane-changing behaviors. The original Nagatani's two lane lattice model [14] is what the new model degenerates into when f=1 and n=2, making Nagatani's two lane lattice model a special case of the model discussed in this paper.

Eliminating the velocity variables in equations (6), (7), the evolution equation of traffic flow density in the muti-lane scenario can be obtained as follows:

(8) $$\rho_{j}\left(t+2\tau \right)-\rho_{j}\left(t+\tau \right)+\tau \rho_{0}^{2}\left[\left(V\left(\rho_{j+1}\left(t\right)\right)-V\left(\rho_{j}\left(t\right)\right)\right)\right]-\gamma \left(n-1\right)\tau \left|\rho_{0}^{2}V^{\prime }\left(\rho_{0}\right)\right|\left[\rho_{j-1}\left(t+\tau \right)+f\rho_{j+1}\left(t+\tau \right)-\left(f+1\right)\rho_{j}\left(t+\tau \right)\right]=0$$

## 3 Linear stability analysis

The steady-state solution of Eq. (8) at the beginning, assuming a uniform traffic flow in muti-lane system with density $\rho_{0}$ and average velocity $V\left(\rho_{0}\right)$ distributed on a circular road, is:

(9) $$\rho_{j}\left(t\right)=\rho_{0}\;，\;v_{j}\left(t\right)=V\left(\rho_{0}\right)$$

In order to analyze the effect of drivers' aggressive lane-changing characteristics on the stability of multi-lane traffic system in a small perturbation scenario, we add a small perturbation signals $y_{j}\left(t\right)$ to the equilibrium traffic flow, i.e.

(10) $$\rho_{j}\left(t\right)=\rho_{0}+y_{j}\left(t\right)$$

Substituting Eq. (10) into the density evolution Eq. (8) and linearizing the equation can be obtained as follows:

(11) $$y_{j}\left(t+2\tau \right)-y_{j}\left(t+\tau \right)-\left(n-1\right)\tau \gamma \left|\rho_{0}^{2}V^{\prime }\left(\rho_{0}\right)\right|\left[y_{j-1}\left(t+\tau \right)+fy_{j+1}\left(t+\tau \right)-\left(f+1\right)y_{j}\left(t+\tau \right)\right]+\tau \rho_{0}^{2}V^{\prime }\left(\rho_{0}\right)\left[y_{j+1}\left(t\right)-y_{j}\left(t\right)\right]=0$$

where $V^{\prime }\left(\rho_{0}\right)=\left[dV\left(\rho_{j}\right)/d\rho_{j}\right]{|}_{\rho_{j}=\rho_{0}}$.Expanding $y_{j}\left(t\right)$ in Eq. (11) as a Fourier series form: $y_{j}\left(t\right)=\exp\left(ikj+zt\right)$, one can obtain:

(12) $$e^{2\tau z}-e^{\tau z}+\tau \rho_{0}^{2}V^{\prime }\left(\rho_{0}\right)\left(e^{ik}-1\right)-\left(n-1\right)\tau \gamma \left|\rho_{0}^{2}V^{\prime }\left(\rho_{0}\right)\right|\left(e^{-ik}-\left(f+1\right)+fe^{ik}\right)e^{\tau z}=0$$

To obtain the first and second order terms with respect to parameter ik, the variable z in Eq. (12) is expanded into the form $z=z_{1}ik+z_{2}{\left(ik\right)}^{2}+\cdots$, from which we get

(13) $$z_{1}=-\rho_{0}^{2}V^{\prime }\left(\rho_{0}\right)+\left(n-1\right)\left(f-1\right)\gamma \left|\rho_{0}^{2}V^{\prime }\left(\rho_{0}\right)\right|$$

(14) $$z_{2}=-\frac{3\tau z_{1}^{2}}{2}-\frac{\rho_{0}^{2}}{2}V^{\prime }\left(\rho_{0}\right)+\frac{1}{2}\gamma \left(1+f\right)\left(n-1\right)\left|\rho_{0}^{2}V^{\prime }\left(\rho_{0}\right)\right|+\gamma \left(f-1\right)\left(n-1\right)\left|\rho_{0}^{2}V^{\prime }\left(\rho_{0}\right)\right|\tau z_{1}$$

If $z_{2}$ is negative, the small perturbation signal will spread and increase within the multi-lane traffic flow, ultimately resulting in instability and traffic congestion, according to the long-wave expansion method criterion. On the other hand, if $z_{2}$ is positive, the traffic flow will return to its initial stable state as the small perturbation signal gradually weakens over time. Consequently, the following are the crucial stability conditions for the multi-lane lattice model that take into account the impact of the driver's aggression when changing lanes:

(15) $$\tau =-\frac{1+\left(1+f\right)\left(n-1\right)\gamma }{\left[3+\left(n-1\right)\left(f-1\right)\gamma \right]\left[1+\left(n-1\right)\left(f-1\right)\gamma \right]\rho_{0}^{2}V^{\prime }\left(\rho_{0}\right)}$$

The criterion for maintaining stability in multi-lane highway traffic flow lattice model is:

(16) $$\tau <-\frac{1+\left(1+f\right)\left(n-1\right)\gamma }{\left[3+\left(n-1\right)\left(f-1\right)\gamma \right]\left[1+\left(n-1\right)\left(f-1\right)\gamma \right]\rho_{0}^{2}V^{\prime }\left(\rho_{0}\right)}$$

Eq. (16) shows that the stability of the multi-lane system is closely related to the number of lanes and the driver's lane-changing aggressiveness characteristics. In particular, when f=1 and n=2, the stability conditions of the new model in this paper degenerate into stability conditions consistent with the Nagatani's two-lane lattice model [14] as follows:

(17) $$\tau <-\frac{1+2\gamma }{3\rho_{0}^{2}V^{\prime }\left(\rho_{0}\right)}$$

**Fig. 2:**
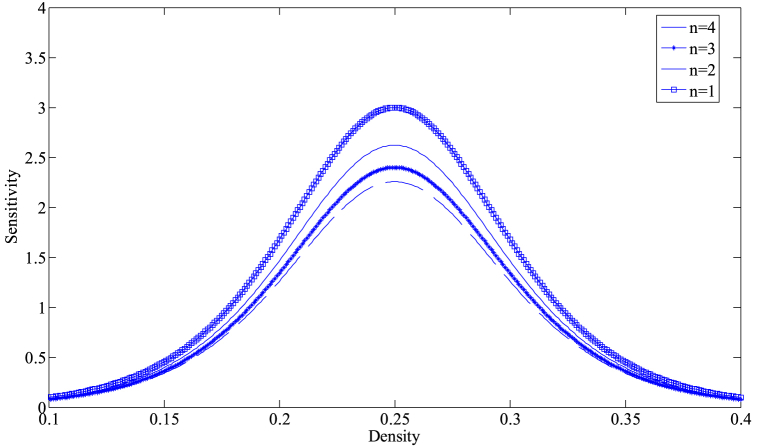
The neutral stability curves for presented model in density-sensitivity space with different *n* when f=2, γ=0.1.

**Fig. 3:**
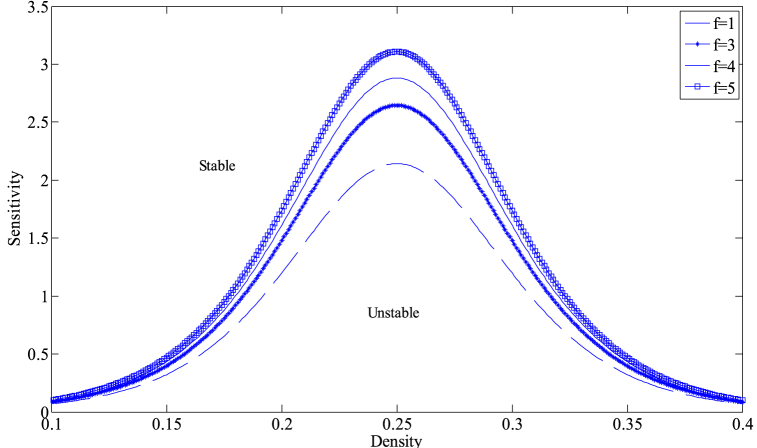
The neutral stability curves for presented model in density-sensitivity space with different f when n=3, γ=0.1.

Based on Eq. (14), Fig. 2 provides the critical stability curves of the proposed model for different numbers of lanes with f=2, γ=0.1. The density-sensitivity space is divided into two parts by each neutral stability curve, where the upper part represents the stability region and the lower part signifies the instability region. The vertex of each curve is known as the critical stabilization point, with its coordinates being $\left(\rho_{c},a_{c}\right)$. From Fig. 2, it can be observed that the corresponding stability region in the phase diagram space gradually expands with the increase in the number of lanes, indicating that increasing the number of lanes in the freeway system is conducive to improving the stability of traffic operation. On the other hand, Fig. 3 depicts the critical stability curves of the new model corresponding to different drivers' lane-changing aggressiveness coefficients at n=3, γ=0.1. As evident from Fig. 3, the stability of the new model improves as the value of f decreases, suggesting that in a multi-lane system, drivers can effectively avoid the obstruction of preceding vehicles by exhibiting more aggressive lane-changing behavior. They can utilize the space in adjacent lanes to their full potential, adjusting their speed to reach an optimal speed sooner, which in turn reduces traffic congestion and improves the stability of the traffic flow.

## 4 Nonlinear analysis for multi-lane highway scenarios

Traffic congestion is a major problem in traffic flow research. Nonlinear density wave equations can help to analyze the formation mechanism and dissipation process of traffic congestion. In this section, we will use nonlinear analysis to study the evolution process of traffic flow near the critical stable point $\left(\rho_{c},a_{c}\right)$ of a multi-lane highway system. To accomplish this, slow variables *X* and *T* are introduced based on the spatial variable *j* and the temporal variable *t*, respectively, and are defined as follows:

(18) $$X=\varepsilon \left(j+bt\right)，\;T=\varepsilon^{3}t,0<\varepsilon \leq 1$$

where parameter *b* is an undetermined quantity, assuming that the density $\rho_{j}\left(t\right)$ satisfies:

(19) $$\rho_{j}\left(t\right)=\rho_{c}+\varepsilon R\left(X,T\right)$$

After carrying out a Taylor expansion up to the order of $\varepsilon^{5}$ and substituting Eqs. (18), (19) into Eq. (8), the partial differential equation can be obtained as follows:

(20) $$\varepsilon^{2}\left[b+\rho_{c}^{2}V^{\prime }+\gamma \left(n-1\right)\left(f-1\right)\rho_{c}^{2}V^{\prime }\right]\partial_{X}R+\varepsilon^{3}\{\frac{3}{2}b^{2}\tau +\frac{1}{2}\rho_{c}^{2}V^{\prime }+\frac{1}{2}\gamma \left(n-1\right)\rho_{c}^{2}V^{\prime }\left[\left(f+1\right)+2b\tau \left(f-1\right)\right]\}\partial_{X}^{2}R+\varepsilon^{4}\{\partial_{T}R+\frac{\rho_{c}^{2}V^{\prime \prime \prime }}{6}\partial_{X}R^{3}+[\frac{7b^{3}\tau^{2}}{6}+\frac{1}{6}\rho_{c}^{2}V^{\prime }+\frac{1}{6}\gamma \left(n-1\right)\rho_{c}^{2}V^{\prime }(3b\tau \left(f+1\right)+\left(3b^{2}\tau^{2}+1\right)\left(f-1\right))]\partial_{X}^{3}R\}+\varepsilon^{5}\left\{[3b\tau +\gamma \left(n-1\right)\rho_{c}^{2}V^{\prime }\left(f-1\right)\tau \right]\partial_{X}\partial_{T}R+[\frac{5b^{4}\tau^{3}}{8}+\frac{1}{24}\rho_{c}^{2}V^{\prime }+\frac{1}{24}\gamma \left(n-1\right)\rho_{c}^{2}V^{\prime }(\left(f+1\right)+4b\tau \left(f-1\right)+6b^{2}\tau^{2}\left(f+1\right)+4b^{3}\tau^{3}\left(f-1\right))]\partial_{X}^{4}R+\frac{1}{12}\rho_{c}^{2}V^{\prime \prime \prime }\partial_{X}^{2}R^{3}\}=0$$

where $V^{\prime }=\left[dV\left(\rho_{j}\right)/d\rho_{j}\right]|\rho_{j}=\rho_{c}$ and $V^{\prime \prime \prime }=\left[d^{3}V\left(\rho_{j}\right)/d\rho_{j}^{3}\right]|\rho_{j}=\rho_{c}$. At the critical point $\left(\rho_{c},a_{c}\right)$, near $\tau =\left(1+\varepsilon^{2}\right)\tau_{c}$, let $b=-\rho_{c}^{2}V^{\prime }-\gamma \left(n-1\right)\left(f-1\right)\rho_{c}^{2}V^{\prime }$, then the second and third order terms of ε in Eq. (20) can be eliminated, resulting in the following simplified equation:

(21) $$\varepsilon^{4}\left[\partial_{T}R-g_{1}\partial_{X}^{3}R+g_{2}\partial_{X}R^{3}\right]+\varepsilon^{5}\left[g_{3}\partial_{X}^{2}R+g_{4}\partial_{X}^{4}R+g_{5}\partial_{X}^{2}R^{3}\right]=0$$

Which is， $g_{1}=-[\frac{7b^{3}\tau_{c}^{2}}{6}+\frac{1}{6}\rho_{c}^{2}V^{\prime }+\frac{1}{6}\gamma \left(n-1\right)\rho_{c}^{2}V^{\prime }(3b\tau_{c}\left(f+1\right)+\left(3b^{2}\tau_{c}^{2}+1\right)\left(f-1\right))]+\left(3b^{2}\tau_{c}^{2}+1\right)\left(f-1\right))]$， $g_{2}=\frac{\rho_{c}^{2}V^{\prime \prime \prime }}{6}$， $g_{3}=\frac{3b^{2}\tau_{c}+2\gamma \left(n-1\right)\left(f-1\right)b\tau_{c}\rho_{c}^{2}V^{\prime }}{2}$，

$$g_{4}=\frac{5b^{4}\tau_{c}^{3}}{8}+\frac{1}{24}\rho_{c}^{2}{V^{\prime }}^{\prime \;}+\frac{1}{24}\gamma \left(n-1\right)\rho_{c}^{2}V^{\prime }\left[\left(f+1\right)+4b\tau_{c}\left(f-1\right)+6b^{2}\tau_{c}^{2}\left(f+1\right)+4b^{3}\tau_{c}^{3}\left(f-1\right)\right]+\left[3b\tau_{c}+\gamma \left(n-1\right)\rho_{c}^{2}V^{\prime }\left(f-1\right)\tau_{c}\right]g_{1}\;g_{5}=\frac{1-\left[6b\tau_{c}+2\gamma \tau_{c}\left(n-1\right)\left(f-1\right)\rho_{c}^{2}V^{\prime }\right]}{12}\rho_{c}^{2}V^{\prime \prime \prime }$$

Introducing the subsequent mathematical transformation:

(22) $$T^{\prime }=g_{1}T,R=\sqrt{\frac{g_{1}}{g_{2}}}R^{\prime }$$

Eq. (21) can be changed by adding correction terms o(ε) to create the mKdV equation.

(23) $$\partial_{T^{\prime }}R^{\prime }-\partial_{X}^{3}R^{\prime }+\partial_{X}{R^{\prime }}^{3}+\varepsilon M\left[R^{\prime }\right]=0$$

where:

(24) $$M\left[R^{\prime }\right]=\frac{1}{g_{1}}\left[g_{3}\partial_{X}^{2}R^{\prime }+g_{4}\partial_{X}^{4}R^{\prime }+\frac{g_{1}g_{5}}{g_{2}}\partial_{X}^{2}{R^{\prime }}^{3}\right]$$

Ignoring the O(ε) term, the kink-antikink solution is given by:

(25) $$R_{0}^{\prime }\left(X,T^{\prime }\right)=\sqrt{c}\tanh\sqrt{\frac{c}{2}}\left(X-cT^{\prime }\right)$$

The propagation speed of traffic density waves close to the critical point $\left(\rho_{c},a_{c}\right)$ is represented by the parameter *c*, which needs to meet the following solvability requirement in order to be calculated:

(26) $$\left(R_{0}^{\prime },M\left[R_{0}^{\prime }\right]\right)=\int_{-\infty }^{+\infty }dXR_{0}^{\prime }\left(X,T^{\prime }\right)M\left[R_{0}^{\prime }\left(X,T^{\prime }\right)\right]=0$$

The calculation formula for the parameter *c* can be obtained based on the method described in Ref. [71] as follows:

(27) $$c=5g_{2}g_{3}/\left(2g_{2}g_{4}-3g_{1}g_{5}\right)$$

Therefore, near the critical point $\left(\rho_{c},a_{c}\right)$, the evolution law of traffic congestion phase transition in multi-lane highway system can be described by the mKdV equation, and its kink-antikink density wave solution is given by:

(28) $$\rho_{j}\left(t\right)=\rho_{c}+\sqrt{\frac{g_{1}c}{g_{2}}\left(\frac{\tau }{\tau_{c}}-1\right)}\tanh\sqrt{\frac{c}{2}\left(\frac{\tau }{\tau_{c}}-1\right)}\left[j+\left(1-cg_{1}\left(\frac{\tau }{\tau_{c}}-1\right)\right)t\right]$$

The corresponding amplitude *A* can be calculated using the formula:

(29) $$A=\sqrt{\frac{g_{1}c}{g_{2}}\left(\frac{\tau }{\tau_{c}}-1\right)}$$

The density wave solution describes the fluctuation phenomenon and the evolution law of traffic flow density in the multi-lane highway system. The nonlinear analysis results show that the traffic congestion flow near the critical point $\left(\rho_{c},a_{c}\right)$ in the multi-lane system propagates in the form of kink-antikink density waves described by the mKdV equation, which is proved by Fig. 5, Fig. 7 in the subsequent numerical simulations in Section 5.

Based on the critical stability condition Eq. (15), we can obtain the value of the critical sensitivity $a_{c}$. Further, utilizing Eq. (29) in the nonlinear analysis, we can calculate the value of the density wave amplitude *A* of the traffic flow. In Table 2, the specific data for the corresponding critical sensitivity coefficients $a_{c}$ and the amplitudes *A* of the proposed model at different values of *f* are listed, where the input parameters are n=3, γ=0.05. From Table 2, we can clearly observe that as the value of *f* decreases, the corresponding values of the critical sensitivity coefficients $a_{c}$ and the amplitudes *A* of the kink-antikink density waves in traffic congestion also decrease accordingly. This result suggests that in a multi-lane traffic system, as the driver's lane-changing aggressiveness increases (i.e., the adjustment intensity coefficient *f* decreases), the fluctuation amplitude of traffic congestion gradually decreases, thereby improving the stability of the traffic flow.

**Table 2:** The critical sensitivity $a_{c}$ and the amplitude *A* of the proposed model for various *f,* where τ=1, γ=0.05, *n* = 3.

*f*	3.5	3.25	3	2.75	2.5	2.25	2	1
α_*c*_	2.8017	2.7724	2.7429	2.7132	2.6833	2.6533	2.6231	2.5000
*A*	0.1572	0.1553	0.1533	0.1514	0.1495	0.1477	0.1459	0.1398

For comparative analysis, under identical conditions, Table 3 presents the variation of critical sensitivity $a_{c}$ and density wave amplitude values *A* for different values of *f* when the proposed model is reduced to the classical two-lane lattice model framework (*n* = 2). From Tables 3 and it can be observed that the effect of the parameter *f* on $a_{c}$ and *A* in the two-lane system exhibits a similar pattern to that shown in Table 2. Specifically, when *f* = 1 in Table 3, the model corresponds to the original Nagatani two-lane lattice model [14], where the drivers' aggressiveness in lane-changing is at its maximum, and the corresponding stability of the traffic system is optimal. Meanwhile, by comparing Table 2, Table 3, it can be noted that the values of $a_{c}$ and *A* in Table 2 are smaller than those in Table 3 for the same parameter *f*. This indicates that, under the same conditions, the three-lane system exhibits greater stability compared to the two-lane system in terms of traffic operation.

**Table 3:** The critical sensitivity $a_{c}$ and the amplitude *A* of the proposed model for various *f,* where τ=1, γ=0.05, n = 2.

*f*	3.5	3.25	3	2.75	2.5	2.25	2	1
α_*c*_	2.8699	2.8558	2.8417	2.8275	2.8133	2.7991	2.7848	2.7273
*A*	0.1546	0.1535	0.1524	0.1514	0.1503	0.1492	0.1481	0.1438

## 5 Numerical simulation

**Fig. 4:**
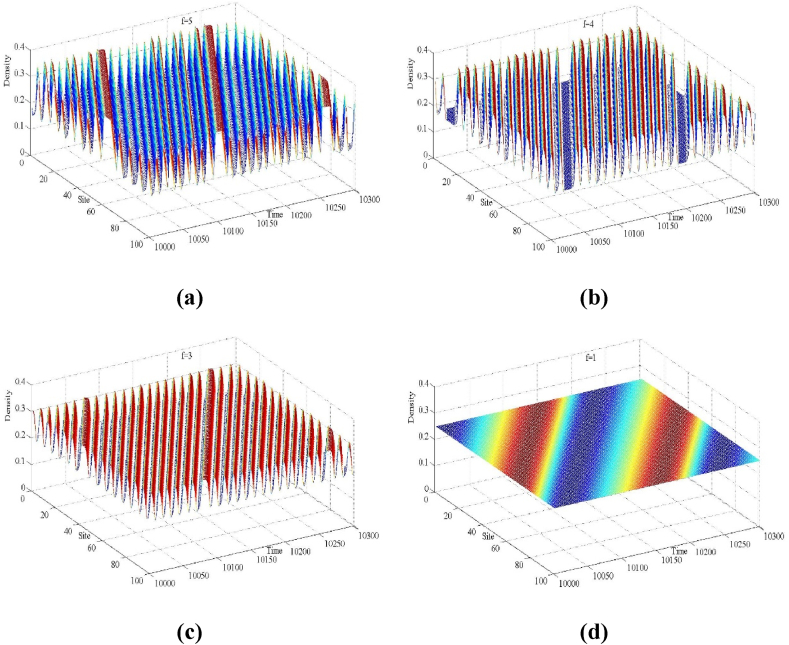
Space-time evolution of the density for (a) f=5, (b) f=4, (c) f=3 and (d) f=1, (a=2.2, n=3).

**Fig. 5:**
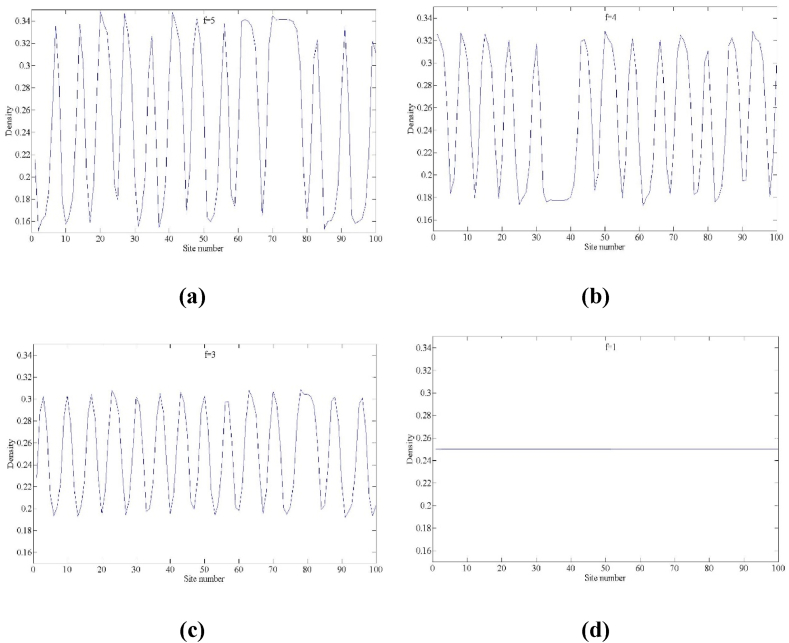
Density profiles of the density wave at t = 10,300 correspond to panels in Fig. 4 respectively.

**Fig. 6:**
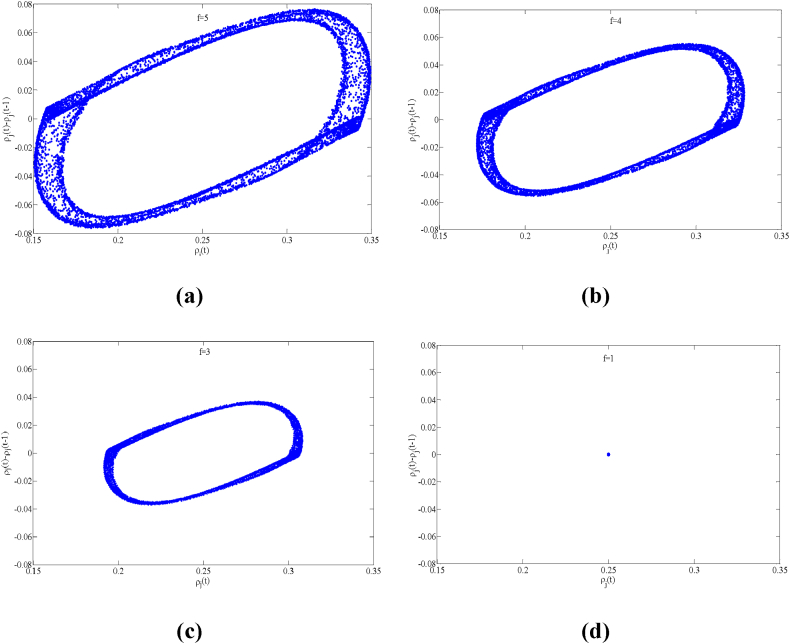
Plot of the density difference $\rho_{j}\left(t\right)-\rho_{j}\left(t-1\right)$ against density $\rho_{j}\left(t\right)$ for t = 15,000–20300 when a=2.2, n=3 for (a) f=5, (b) f=4, (c) f=3 and (d) f=1, respectively.

**Fig. 7:**
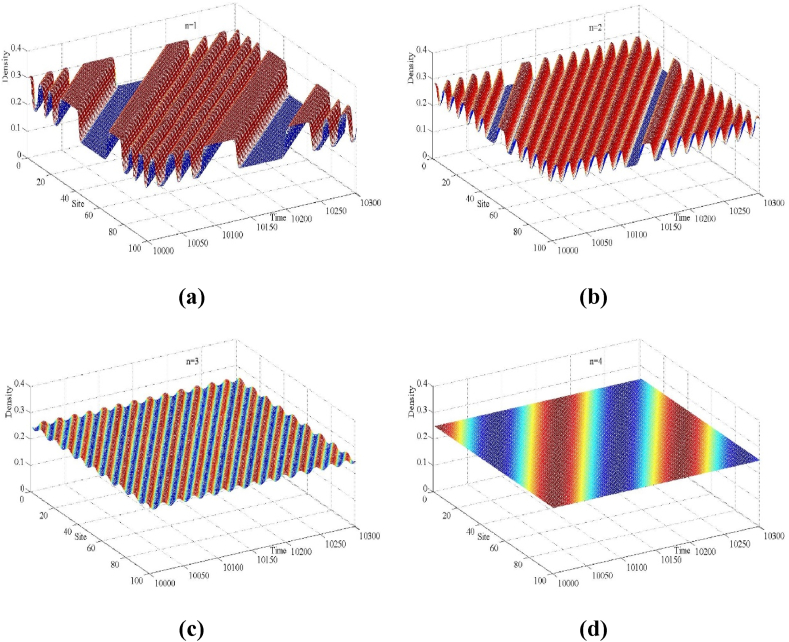
Space-time evolution of the density for (a) n=1, (b) n=2, (c) n=3 and (d) n=4, (a=2.38, f=2).

**Fig. 8:**
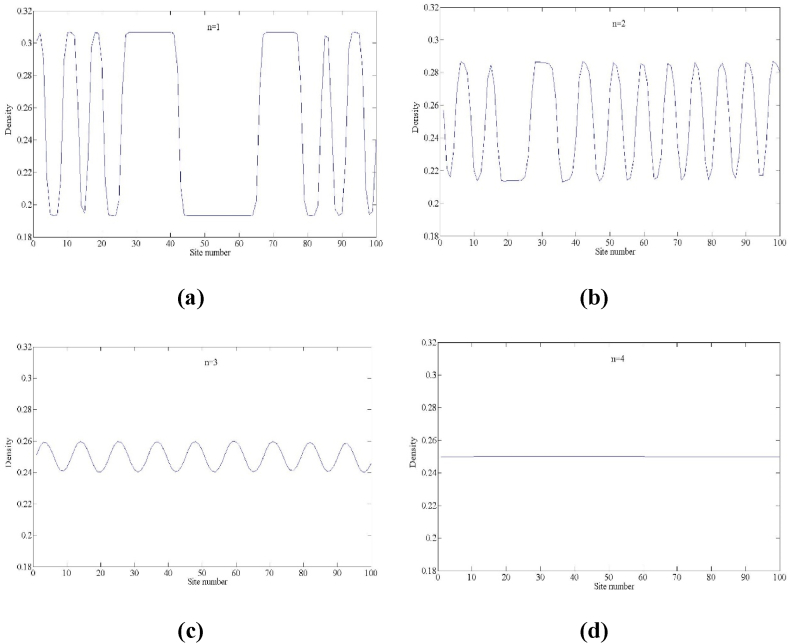
Density profiles of the density wave at t = 10,300 correspond to panels in Fig. 7 respectively.

**Fig. 9:**
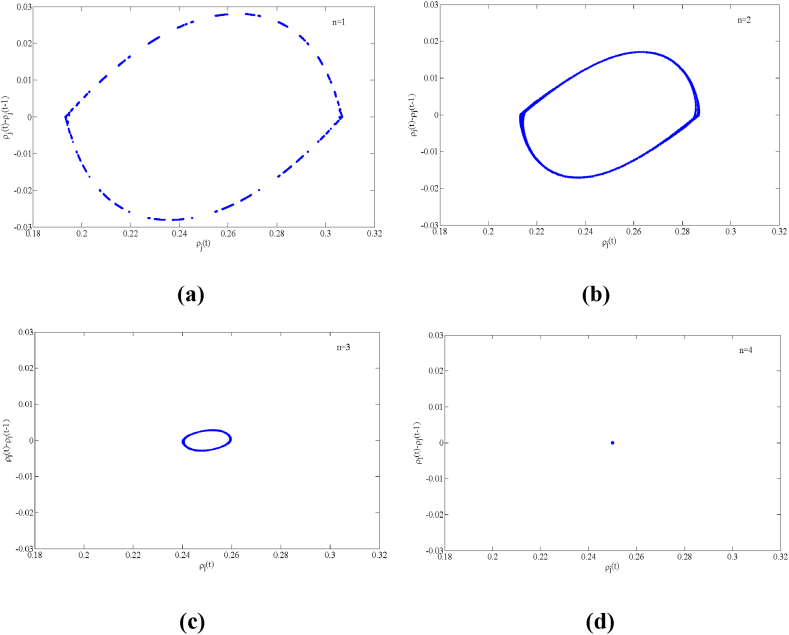
Plot of the density difference $\rho_{j}\left(t\right)-\rho_{j}\left(t-1\right)$ against density $\rho_{j}\left(t\right)$ for t = 15,000–20300 when a=2.38, f=2 for (a) n=1, (b) n=2, (c) n=3 and (d) n=4, respectively.

**Fig. 10:**
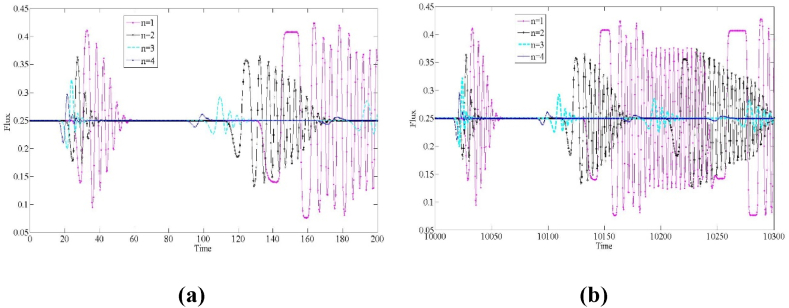
The flux evolution of 25th lattice site with a=2.38, f=3.

**Fig. 11:**
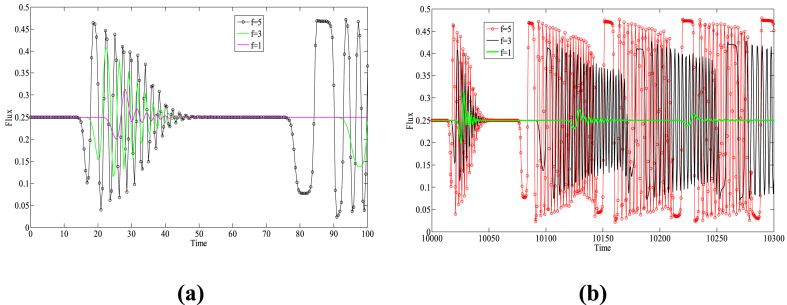
The flux evolution of 25th lattice site with a=2.2, n=3.

**Fig. 12:**
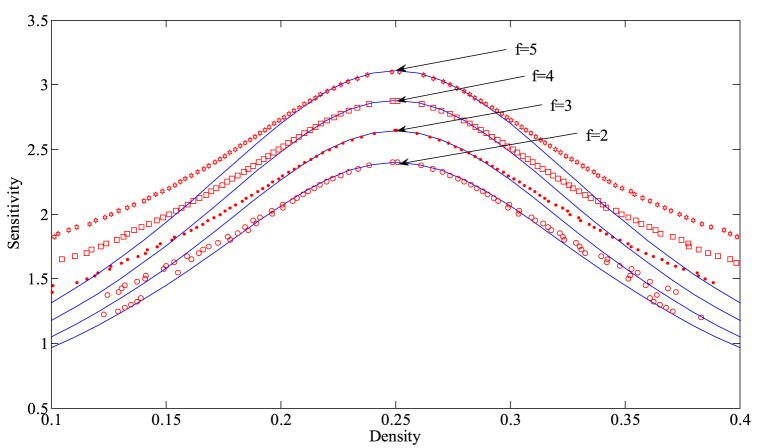
The coexisting curves in density-sensitivity space with different *f* when n=3, γ=0.1.The blue solid line indicates the analytical results obtained through the mKdV equation. The curves composed of red markers represent the corresponding simulation results.

**Fig. 13:**
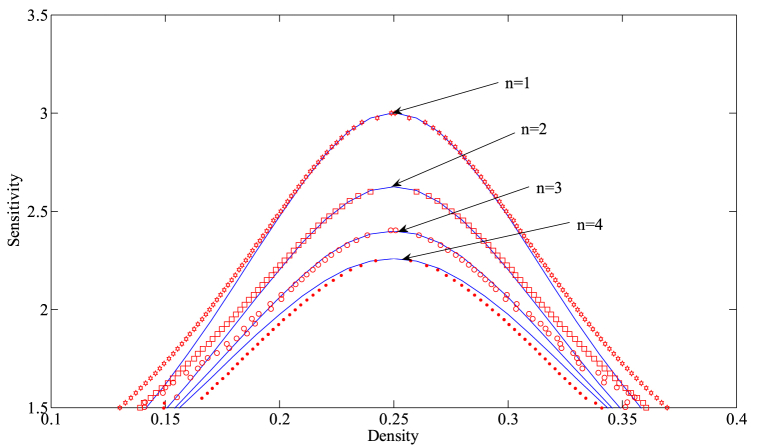
The coexisting curves in density-sensitivity space with different *n* when f=2, γ=0.1.The blue solid line indicates the analytical results obtained through the mKdV equation. The curves composed of red markers represent the corresponding simulation results.

Numerical simulations for the density evolution equation of the multi-lane lattice model for highway system, represented by Eq. (8), were carried out in this section. Periodic boundary conditions were applied, and the following initial states were established:

$\rho_{j}\left(0\right)=\rho_{0}=0.25$, $\rho_{j}\left(1\right)=\rho_{j}\left(0\right)=0.25$, j≠50,51, $\rho_{j}\left(1\right)=0.25+0.1$ (j=50)， $\rho_{j}\left(1\right)=0.25-0.1$ (j=51). The input parameters were defined as follows: total number of road cells N=100，lane changing rate γ=0.1. The simulation results are illustrated in Fig. 4, Fig. 5, Fig. 6, Fig. 7, Fig. 8, Fig. 9, Fig. 10, Fig. 11, Fig. 12, Fig. 13.

Fig. 4 shows the three-dimensional evolution of the traffic density for the proposed model after t = 10,000 time steps, corresponding to the lane-changing aggressiveness coefficient f. The settings of the simulation's input parameters are a=2.2, n=3.When f=3,4,5, which corresponds to subfigures (a), (b), and (c), respectively, due to the fact that the stability condition (16) is not satisfied, the small perturbation signals imposed in the uniform traffic flow are gradually amplified in the multi-lane highway with the development of time and eventually develop into a stop-and-go traffic congestion phenomenon. By comparing the fluctuation amplitude of traffic density in subfigures (a), (b), and (d), it can be found that the fluctuation amplitude decreases gradually as the parameter decreases. In particular, when the parameter is set f=1, the traffic stability condition (16) is satisfied, at which time the small disturbance signal completely disappears in the traffic flow and all the traffic flows in the road system return to the equilibrium state.

In correspondence to Figs. 4 and 5 is provided to further illustrate the distribution of traffic density in the multi-lane highway for all lattice points at time step t = 10,300, demonstrating the evolution characteristics of traffic density waves in the presence of drivers' lane-changing aggressiveness effects. From Fig. 5, it can be found that traffic congestion in the multi-lane highway system appears as a kink-antikink density wave, and the amplitude of the wave decreases sharply with the decrease in the lane-changing aggressiveness coefficient *f*. Furthermore, in Fig. (d), when *f* = 1, the traffic density distribution is consistent across all lattice point locations, and the traffic flow reverts to the initial steady state.

To more visually illustrate how the aggressive lane-changing strategy adopted by drivers in a multi-lane system affects the stability of traffic flow, Fig. 6 depicts a plot of the density difference $\rho_{j}\left(t\right)-\rho_{j}\left(t-1\right)$ versus the density $\rho_{j}\left(t\right)$ of the new model for a specific time period (t = 15,000 to 20,300) when the parameter f takes different values. The collection of these data points ($\rho_{j}\left(t\right)-\rho_{j}\left(t-1\right)$, $\rho_{j}\left(t\right)$) forms a hysteresis loop on the graph. The size of the area enclosed by the hysteresis loop visually represents the stability of the traffic operation: the smaller the area, the more stable the traffic operation.

From Fig. 6, it can be clearly observed that the area of the hysteresis loop is significantly reduced as the value of f gradually decreases from a larger value, i.e., from Fig. (a) to Fig. (d). It is particularly noteworthy that when the value of f is reduced to 1, the hysteresis loop in Fig. 6(d) almost degenerates to a point, as the linear stability condition (16) is satisfied at this point. This indicates that the traffic flow in the system reaches a steady operation state. Thus, as the degree of drivers' aggressive lane-changing increases, the traffic stability performance is significantly improved. Therefore, reasonable driving behavior adjustment is crucial for maintaining smooth road traffic.

In conclusion, the traffic flow evolution processes in Fig. 4, Fig. 5, Fig. 6 consistently show that in the multi-lane highway system, aggressive lane changing behavior by drivers has a significant positive effect on the stability of the traffic flow and a good inhibition effect on the formation of traffic congestion, and this numerical simulation conclusion is consistent with the theoretical analysis results of linear stability.

Fig. 7 exhibits the three-dimensional evolution of the traffic density for the new model at *t* = 10,000 time steps corresponding to the number of lanes n=1,2,3,4, where the simulation input parameters are a=2.38, f=2. Corresponding to Figs. 7 and 8 shows the traffic density distribution at all lattice sites at t = 10,300 time steps (t = 10,300). From the traffic evolution depicted in Fig. 7, Fig. 8, it can be observed that when the traffic stability condition (16) is not satisfied, small perturbation signals in the multi-lane highway traffic flow gradually evolve and develop into traffic congestion. This congestion propagates upstream along the traffic flow and manifests as a kink-antikink density wave formation, where sparse low-density traffic flows alternate with aggregated high-density clusters. However, generally speaking, it is evident that with an increase in the number of lanes n, the fluctuation amplitude of the traffic flow becomes significantly smaller. The mutual conversion amplitude between the free-flow phase and the blocking phase decreases, resulting in a smoother traffic flow. In particular, when the number of lanes is n=4, the traffic flow in the multi-lane highway system returns to the steady state of uniform motion, as the stability condition (16) is satisfied and the perturbation signal disappears completely. These simulation results are in perfect agreement with the findings of the linear stability theory analysis presented in Section 3.

Fig. 9 gives a plot of the density difference variable $\rho_{j}\left(t\right)-\rho_{j}\left(t-1\right)$ versus the density variable $\rho_{j}\left(t\right)$ of the new model at time t = 15,000–20300 when the number of lanes *n* takes different values. From the figure, it can be found that the area of the hysteresis loop is getting smaller and smaller as the number of lanes in the multi-lane system increases, which means that the stability of the traffic flow is enhanced with the increase of the number of lanes, and the fluctuation of the traffic flow is getting smaller and smaller, especially when the number of lanes *n* = 4, since the stability (16) is satisfied, corresponding to Fig. 9(d), the traffic flow is the same density at each time step (the density difference between two consecutive time steps is zero). At this point, the traffic flow of the traffic system operates in steady state.

Keeping the traffic flux at a high volume is one of the main goals pursued by modern intelligent transportation. Fig. 10 explores the evolutionary distribution of traffic flux at the 25th lattice site at different numbers of lanes (n=1,2,3,4), where the input parameters are a=2.38, f=3, γ=0.1. It can be found that during the initial 200 time steps, the small perturbation signal propagates and amplifies rapidly on the roadway, and the traffic volume undergoes sharp fluctuations. With the development of time, at t = 10,000–10300, the traffic flux fluctuates and changes in more time periods, and the disturbed wave and the traffic volume undergoes tidal changes in a large range of time steps. However, it can be found that, with the increase of the number of lanes n, the number of time periods in which significant changes in the flow of vehicles occur is significantly smaller, and the flow fluctuates in a smaller interval, which indicates that the multi-lane system is beneficial to the flow of vehicles for a long period of time to maintain a more stable level of traffic through the vehicle, and the adverse effects of the small perturbation signals can be confined to a much smaller time range.

Fig. 11 shows the evolution of the traffic flow distribution in the first 100 time steps and within t = 10,000–10300 time steps at different driver lane-changing aggressiveness coefficients (f=1,3,5). From Fig. 11, it can be found that the fluctuation interval of the traffic flow gradually narrows as the lane-changing aggressiveness coefficient f increases, especially when f=1, at that time, the traffic flow in the multi-lane highway system develops over time with the influence of small perturbation signals gradually decreasing, which allows the traffic flow to be maintained at a more stable flow level for a long period of time, with weak fluctuations occurring only within a few small time slices. This significantly indicates that, based on the multi-lane system, drivers can make use of the lane-changing behavior to keep the traffic flow at a high level, and as the degree of aggressiveness of the driver's lane changing increases, the stability of the traffic flow is gradually enhanced, and the traffic flux can be maintained at a high level for a long period of time. Thus, the popularization of multi-lane systems and the increase of drivers' aggressive lane changing are of great practical significance for optimizing the efficiency of road traffic.

In summary, the simulation results presented in Fig. 4, Fig. 5, Fig. 6, Fig. 7, Fig. 8, Fig. 9, Fig. 10, Fig. 11 are not only highly consistent with the linear stability theory results in Section 3, but they also empirically demonstrate the significant effects of drivers' aggressive lane-changing rules and the number of lanes on the flow dynamics characteristics of multi-lane systems. These findings suggest that drivers adopting relatively aggressive lane-changing strategies in multi-lane traffic systems can effectively contribute to the stability of the traffic flow and optimize the overall traffic efficiency. Furthermore, an increase in the number of lanes similarly contributes positively to the stability of the traffic system. These findings provide important guidance for transportation planning and management authorities. By optimizing road design, adjusting the number of lanes, and increasing training for drivers' maneuvering skills, the operational efficiency of the road system can be significantly improved, creating a more efficient traveling environment.

The primary function of nonlinear analysis in Section 4 is to accurately depict the dynamic evolution of traffic flow near the critical point of the traffic system, providing us with a theoretical tool for a thorough examination of the evolution law of traffic congestion. By using the coexistence curve of the new model as an object, we compare the numerical simulation results with the theoretical results of Eq. (28) to confirm the consistency between the theoretical outcomes of the nonlinear analysis and the numerical simulation results. The pertinent results are displayed in Fig. 12, Fig. 13. Fig. 12, Fig. 13 show the coexistence curves of the new model for different values of the adjustment intensity coefficient *f* and the number of lanes *n*, respectively. In these two figures, the blue solid line represents the theoretical analytical results of the kink-antikink density wave solution obtained based on the mKdV equation (Eq. (28)), while the curves formed by the red marked points represent the numerical simulation results.

As can be seen from the figures, when approaching the critical point of the system, the red curve aligns almost perfectly with the corresponding blue curve, indicating that the mKdV equation is able to accurately describe the evolution of the traffic flow in the vicinity of the critical point. However, as we move further away from the critical point, the two curves gradually deviate from each other. This is due to the fact that the mKdV equation is primarily used to capture the dynamics near the critical point, and its descriptive ability diminishes in regions distant from the critical point.

This phenomenon not only verifies the effectiveness of the nonlinear analysis theory near the critical point of the system but also demonstrates its accuracy in portraying the propagation law of traffic congestion. At the same time, it also provides a robust method to verify the accuracy and reliability of numerical simulation results.

## 6 Conclusion

The existing traffic flow lattice model has a very simple lane-changing mechanism and is not fully compatible with the actual driving process. In order to make the theory of multi-lane highway traffic flow lattice model closer to traffic reality, in this paper, for the multi-lane scenario of a highway, we introduce the parameter that characterizes the aggressiveness of the driver's lane-changing in real traffic, redesign the lane-changing mechanism of the multi-lane system, and on the basis of this, we propose a new multi-lane lattice model. The linear stability condition of the new model is derived analytically using linear stability theory. Based on the nonlinear analysis process for the new model, the density wave equation describing the evolution law of traffic congestion near the critical stability point of the system is obtained. In order to verify the correctness of the theoretical analysis, numerical simulations are carried out under periodic boundary conditions. The numerical simulation results are in perfect agreement with the theoretical analysis. The analysis results show that the stability of traffic flow gradually increases with the increase in the number of lanes in the multi-lane system. At the same time, with the increased aggressiveness of the driver's lane-changing, the traffic congestion in the multi-lane highway system is effectively suppressed, and the degree of traffic flow fluctuation is gradually reduced. In addition, the traffic simulation results of the new model also show that the number of lanes of the multi-lane system and the driver aggressiveness lane-changing characteristics are closely related to the traffic flux of the road traffic system and the time level of the system to maintain the optimal traffic flux, which is specifically manifested in the fact that the increase in the number of lanes and the increase in the level of the driver aggressiveness lane-changing can make the traffic flow remain at the optimal traffic flow for a long period of time and effectively reduce the frequency and amplitude of the traffic flow fluctuation. It can be seen that in today's increasingly serious traffic congestion, the moderate development of multi-lane systems and the adoption of certain strategies to improve the level of driver lane-change aggressiveness are of great practical significance for optimizing road traffic efficiency.

Nevertheless, the model developed in this paper has limitations in simulating the operating laws of mixed traffic flow composed of multiple vehicle types because it only focuses on a single vehicle type and hasn't taken into account the traffic flow scenario in which multiple vehicle types are mixed. In order to expand and enhance the analysis of this model and improve our ability to simulate the operating laws of mixed traffic flow scenarios, we intend to incorporate additional vehicle types in subsequent work. In addition, although the model takes into account aggressive lane-changing behavior by drivers, there is no detailed discussion of the potential impact of different driving behaviors on the model results. We extend the model in future work to explore the diversity of driving behaviors.

## Data availability

No data was used for the research described in the article.

## Ethics declarations

Informed consent was not required for this study because this study does not involve clinical drugs, animal experiments, race and other ethical issues.

## CRediT authorship contribution statement

**Yi-rong Kang:** Writing – original draft, Validation. **Chuan Tian:** Visualization, Supervision, Project administration, Methodology.

## Declaration of competing interest

The authors declare that there is no conflict of interests regarding the publication of this paper.
